# Causes and contributing factors of maternal mortality in Bosaso District of Somalia. A retrospective study of 30 cases using a Verbal Autopsy approach

**DOI:** 10.1080/16549716.2019.1672314

**Published:** 2019-10-10

**Authors:** Jamila Ahmed Aden, Hinda Jama Ahmed, Per-Olof Östergren

**Affiliations:** a Faculty of Medicine and Health Sciences, East Africa University, Bosaso, Puntland State of Somalia, Somalia; b Social Medicine and Global Health, Department of Clinical Sciences Malmö, Lund University, Lund, Sweden; c Department of Epidemiology and Global Health, Umeå University, Umeå, Sweden

**Keywords:** Maternal health, maternal mortality in Somalia, causes and contributing factors, Three Delay Model, Verbal autopsy

## Abstract

**Background**: Somali women suffer from one of the highest maternal mortality rates in the world. Somalia characterises a specific low-income country situation with a mix of newly urbanized and nomadic culture combined with a frail health care infrastructure set in a post-conflict era. Very little is known about the effects that these contextual factors can have on maternal mortality.

**Objectives**: To explore and describe causes and contributing factors concerning maternal deaths in the Bosaso District, Puntland State of Somalia.

**Methods:** Data was collected using an adapted Verbal Autopsy tool. In 2017 30 cases of maternal deaths occurring in 2016 in the Bosaso District were reviewed. Information was assessed by three independent reviewers who classified the cause of death and the contributing factors. The Three Delay Model was employed to identify socio-cultural and economic and health system factors that may have contributed to these maternal deaths.

**Results**: Direct obstetric deaths accounted for 28 cases. Among these, haemorrhage was the leading cause, followed by eclampsia, sepsis and obstructed labour. Two cases were indirect obstetric deaths, caused by anaemia. All three types of delay were frequent among the studied cases. Delay in deciding to seek care was found in 25 cases, delay in reaching care in 22 cases and delay in receiving health care in 24 cases. Lack of knowledge, money, transportation, poor access and availability of adequate services, as well as substandard management by health care providers, were all underlying the delays.

**Conclusion**: A comprehensive intervention programme is needed in order to decrease maternal mortality among Somali women. Such a programme must include health education, improved referral systems and strategic upgrading of care services.

## Background

Globally, the majority (99%) of all maternal deaths take place in middle and low income countries [1]. Of these, over half occur in Sub-Saharan Africa. The vast gap in maternal mortality between the high-income countries and middle- and low-income countries illustrates that a great majority of the maternal deaths that occur in low-income countries are preventable given timely and adequate intervention [1]. The Millennium Development Goal 5, target set a three quarters reduction in maternal mortality for the period 1990 to 2015 [2]. The global maternal mortality ratio was in fact reduced by almost half from 515 per 100,000 live births in 1990 to 303 in 2015 [1]. In the 2014 annual global report by the Save the Children Fund, ranked Somalia at the very bottom out of 178 countries. It can be said therefore that Somalia is the worst place in the world to become a mother. The Maternal Mortality Rate in Somalia in 2016 was 732 maternal deaths per 100,000 live births, which is among the highest in the world, albeit this fell from 1,044 in 2012, according to the latest estimates of Somalia Health Indicators Report [3]. The Total Fertility Rate in Somalia according to a UNICEF report in 2016 is 6.6 per woman, this being the third highest rate in the world [3]. Accordingly, the lifetime risk of maternal death in Somalia is among the world’s highest, 1 in 22, exceeded by only Chad and Sierra Leone [3].

In Somalia, a vital registration system is lacking to review maternal deaths. As a result, adequate information on the extent and underlying factors of maternal deaths is not available for use by the government or other agencies in developing policies and effective interventions. Currently, the health facilities record and classify maternal deaths solely by medical causes. This may conceal important information for understanding the underlying causes and determinants of maternal deaths. For example, information is needed on the route and actions taken by the deceased woman prior to her arrival at the health facility, which will offer clues about possible physical, socio-cultural and economic barriers that impede access to appropriate care in a timely manner. Therefore, a detailed study of cases needs to be conducted following the ‘road to death concept’ (referring to the series of events and their underlying circumstances, occurring during the care seeking process, resulting in the death of the mother if not adequate). Furthermore, it may raise awareness among health professionals about the most important factors in the facilities and in the community, which if avoided, could prevent maternal deaths and may stimulate action to address those factors.

One of the main strategies to reduce maternal mortality is providing adequate medical care in obstetric emergencies in a timely manner [4]. Thaddeus and Maine^,^s Three Delays Model (delays in the decision to seek care, in reaching care, and in receiving care) was developed more than 20 years ago for evaluating the circumstances surrounding access to adequate emergency obstetric care [5]. A number of studies have used the model to identify barriers and potential points of intervention to access appropriate emergency obstetric care [6].

This study uses the Verbal Autopsy (VA) technique, which is a widely used approach due to the substantial benefits it offers as a tool in countries such as Somalia, where there is no system of vital registration and the practice of the medical certification of death is non-existent [7]. The VA methodology was introduced in the 1980s and has become a well-utilized and well-validated method in settings lacking other routines for determining cause of deaths [8]. VA is now recommended by WHO as the method of choice in such situations and has published well-validated guidelines [9]. Recently, the VA tool has been further developed into the InterVA-4 version, which provides an increased opportunity to obtain information about emergency obstetric care [10].

The aim of this study is to identify and describe causes contributing to maternal deaths in Bosaso District, Puntland State of Somalia and to further explore the socio-cultural and economic and health service factors in connection with those deaths.

## Methods

### Study setting

The study was performed in Bosaso District, Administrative Divisions of Bari region with an estimated population of 719,512 according to population estimation survey in 2014 of Somalia. It is situated in the North-eastern part of the country on the coast of the Gulf of Aden. Bosaso city is the densest city in Puntland State of Somalia. The estimated total population uptake for Bosaso health facilities was 448,009 in the year 2017 according to Puntland Ministry of Health report. The city is home to the only public district hospital in the region which serves as a referral hospital for all of the four maternal health centres (MCH) that provide basic obstetric care. The hospital, also receives a considerable number of patients from all the other districts in the region.

### Study design and subjects

The study was designed as a cross-sectional assessment of maternal deaths that could be retrieved using records at a defined set of health facilities during a defined period of time. The target population consisted of women who died while pregnant, during delivery, or up to 42 days after delivery between the time 1 March 2016 and 30 March 2017 and who resided within Bosaso District during their last pregnancy until death. The death must have occurred in a health facility (hospital, health centre, health posts), in the community or en route to a health facility.

This resulted in the identification of thirty cases of maternal mortality according to the World Health Organisation International Classification of Diseases version 10 (WHO ICD 10), from the health records in the hospital, three MCH and five traditional birth attendants (TBA).

### Data collection

Data were collected in 2017 by a VA approach regarding 30 cases of maternal deaths during the previous year in Bosaso District. The study used data from records from the mentioned types of health facilities, in addition to VA interviews, which were conducted with the communities; Families and relatives (husband, sisters, mother in law) neighbours, TBAs, community health workers (CHW) and others who had knowledge of the particular cases were interviewed. A recall period between one to 30 weeks was applied, this being well within the recommended range of not more than five years [11]. One main interviewer was used at all times to increase the reliability of the collected information. The ‘*road to death*’ concept (referring to the series of events and their underlying circumstances, occurring during the care seeking process, resulting in the death of the mother if not adequate) was followed in a quest to generate adequate information [12].

A pre-validated Verbal Autopsy Questionnaire (VAQ) was used that contains sections of structured questions to probe for specific signs and symptoms and the background characteristics of the deceased. It also included a section for open ended questions to probe for relatives’ account of events around the woman’s illness and death and symptoms developed during pregnancy, labour, and within six weeks after delivery [13].

A classification form developed by WHO guidelines for Maternal Death Review was used for our VA [12]. This tool was used to review and assign the medical cause of death and the contributing factors. The form consists of two main aspects of maternal death: the first is used for the cause of death (either direct or indirect cause of maternal death) and the second aspect is a checklist, which looks at the contributing aspect of the death.

### Classification by reviewers

All 30 cases were reviewed by three obstetricians using the standard classification form. Information on medical cause of death and the contributing factors was added. The reviews were based on the information collected through the VAQ and the deceased’s health facility records (case notes). The medical cause of death assigned to each case was accepted when two of the reviewers agreed upon a main cause of death and if the three reviewers did not agree then the cause of death was reported as ‘unknown’.

### Data analysis

The R statistical packages was used for descriptive analysis of the quantitative data with regard to causes and contributing factors. Qualitative ínformation was analysed using Open Code software and was coded and allocated according to different categories in the ‘Three Phase Delay’ Model. Finally, the categories were put into a framework for the analysis.

## Results

Table 1 shows the socioeconomic and demographic characteristics of the women including pregnancy and birth outcome. The average age of the deceased women was 31.1 years (SD =  8.4). The youngest among the deceased was 15 years while the oldest was 50 year. Of the 30 cases, only seven (23.3%) had attended formal schooling of which three had completed secondary school. The majority of the women 23 (76.7%) had no education at all or had only attended basic Islamic school (Madrasah). In contrast, 23 of the husbands (76.7) had some formal education. All of the women were married and most of them were their husband’s only wife. More than half of the women (56.7%) were housewives. The husband’s work varied across a wide range of occupations.

**Table 1.** Sociodemographic characteristics and pregnancy details of 30 cases of maternal death in Bosaso District, Puntland State, Somalia.

	N (%)		Ns (%)
***Age***		***Parity***	
15 – 20	5 (16.7)	1–3	6 (20.0)
21 – 25	1 (3.3)	4–6	9 (30.0)
26 – 30	8 (26.7)	7–9	7 (23.3)
31 – 35	8 (26.7)	≥ 10	8 (26.7)
36 – 40	6 (20.0)	***Place of death***	
41 – 45	1 (3.3)	Home	18 (60.0)
46 – 50	1 (3.3)	Hospital	9 (30.0)
***Woman’s education***		Health centre	1 (3.33)
None	6 (20.0)	Private hospital	1 (3.33)
Madrasah	17 (56.7)	En route	1 (3.33)
Primary	4 (13.3)	***Time of the death***	
High School	3 (10.0)	Ante-partum	1 (3.3)
***Husband’s education***		Intra-partum	3 (10.0)
Madrasah	7 (23.3)	Postpartum	26 (86.7)
Primary	7 (23.7(23.3)3)	***Place of delivery***	
High School	13 (43.3)	Home	19 (73.1)
Technical School	3 (10.1)	Hospital	5 (19.3)
***Working for income***		MCH	1 (3.8)
Yes	13 (43.3)	Private hospital	1 (3.8)
No	17 (56.7)	***Delivery outcome***	
***Married***		Stillborne child in singleton pregnancy	16 (61.5)
Yes	30 (100)	Live birth in singleton pregnancy	7 (27.0)
No	0 (0)	Live birth in twin pregnacy	4 (3.8)*
***Husband’s occupation***		Stillborn child in twin pregnancy	2 (7.7)
Shop/store owner/manager	2 (6.7)		
Labourer/Informal sector	3 (10.0)		
Constriction industry	5 (16.7)		
Soldier	3 (10.0)		
Teacher	3 (10.0)		
Transportation	3 (10.0)		
Technical	6 (20.0)		
Farmer	1 (3.3)		
Government/Formal sector	1 (3.3)		
Food Catering Business	3 (10.0)		

*The four live births belong to three pregnancies.

### Pregnancy and birth characteristics of the cases

One third of the women made no antenatal care visits. The twenty women who attended prenatal care had on average two antenatal care visits during their last pregnancy (range = 0–5, SD = 2). Fifteen of the women (50.0%) had seven or more pregnancies. The average number of pregnancies was 7.1 (range =  1–17, SD =  4.3).

### Outcome of the pregnancy

The majority (18) of the mothers died in their home. Looking at the time of death, 26 (86.6%) died after delivery and more than half of those deaths occurred between 1 and 42 days after delivery. Nineteen (73.1%) of the women who died postpartum, delivered at home assisted by a TBA. There were three multiple pregnancies (twins) among the deliveries. However, none of them was diagnosed before delivery. Two of these mothers made more than one antenatal care visits. There were only 11 live births, four of which were from three twin pregnancies. Four women died before they had delivered while the child died as well as the mother in the remaining 18 cases.

### Medical causes of direct and indirect obstetric deaths

Table 2 presents direct and indirect deaths, according to the causes determined by the three reviewers. Twenty-eight (93.3%) of the cases were classified as direct obstetric deaths. Haemorrhage was the most common main cause of death in this group, accounting for (43.3%) deaths. In addition, a further five cases had haemorrhage as an underlying cause of death and if added to the 13 cases where haemorrhage was the main cause of death, this resulted in 18 cases (not in the table). In three cases, the main cause of death was sepsis and in another three cases sepsis was determined as the underlining cause of death. (not in the table)

**Table 2.** Causes of maternal death among 30 women in Bosaso District, Puntland State, Somalia.

	*Ns*	%
**Direct obstetric deaths**		
Haemorrhage	13	43.3
Antepartum	*1*	
Intrapartum	*2*	
Postpartum	*10*	
Eclampsia	8	26.7
Obstructed labour	4	13.3
Sepsis	3	10.0
*Subtotal*	28	93.3
**Indirect obstetric deaths**		
Anaemia	2	6.7

### Healthcare seeking process

All of the cases sought healthcare from different providers (Table 3). There were 16 cases (53.3%) for whom the first place to seek care was from a TBA. Only three cases sought care at only one provider. Reaching a medical facility did not always mean that a woman received the treatment necessary to save her life. In this study 14 of the cases visited as many as three healthcare providers. This indicates that 27 (90.0%) of the women presented to centres where they did not receive the required services. Ambulance service was not available for the majority of these women (92.6%). Hence, the woman and her relatives had to arrange their own means of transportation by hiring a vehicle to take them to the facilities to which they had been referred (This information is not included in the table).

**Table 3.** Sequence of health care seeking among 30 cases of maternal death in Bosaso District, Puntland State, Somalia.

	First contacted health care provider	Second contacted health care provider	Third contacted health care provider
Hospital	0	12	4
Private hospital	0	1	4
Pharmacy	5	1	0
Community/Village health worker	2	2	2
Maternal and child health centre	7	7	2
Traditional birth attendant	16	3	2
Spiritual healer	0	1	0
No contact	0	3	16

### Funds to purchase medical care

The average expenditure on medical care was 79.6 USD ranging between zero to 358 USD (This information is not in the table). Regardless of the blood being donated by relatives or outside people, this service costs patients 40 USD covering the price of the blood bag container and the testing of the blood group.

### Contributing factors

Table 4 shows frequency of contributing factors as per reviewer. Three independent reviewers identified the most frequent factors that caused maternal deaths. Delay in the decision making process and getting to see professional health staff instead of TBAs or CHWs or even pharmacy staff as well as, the lack of knowledge of treatment possibilities were the most common contributing factors.

**Table 4.** Ranking order of contributing factors among 30 cases of maternal death as per reviewer in Bosaso District, Puntland State, Somalia.

	Number of cases in which factor contributed		
	Reviewer		
**Ranking order of contributing factors**	1	2	3
1. Delay in decision making process	30	30	30
2. Delay in getting to see professional health staff	30	30	29
3. Lack of knowledge of treatment possibilities	29	30	29
4. Delay in reaching medical facility	30	28	29
5. Not recognizing severity of the problem	30	28	28
6. Substandard primary care	28	30	28
7. Obstruction in getting care	29	29	27
8. Substandard obstetric referral procedure	28	30	24
9. People essential for decision making not available	26	25	27
10. Disagreement in decision making	20	23	13
11. Lack of transport	19	19	12
12. Lack of money	20	14	16
13. Different perception of the condition	9	7	9

**Table 5.** Determined cause, reported symptoms and experienced delays among 30 verbally autopsied cases of maternal death in Bosaso District, Puntland State, Somalia.

Case #	Cause	Reported symptoms	Experienced delays		
			*Delay 1 (25)*	*Delay 2 (22)*	*Delay 3 (24)*
1	Haemorrhage and sepsis	Bleeding, shortness of breath and offensive vaginal discharge.	Delivered at home by a TBA. Decided to seek medical help 6 days after recognition of the complication. Difficult for the husband to pay $150, family members helped	Seeking care at different providers including spiritual healer. Visited TB clinic twice and was referred second time to hospital	Needed blood transfusion, which was not available, so relatives donated blood, mother in law paid for the testing and bags.
2	Haemorrhage and retained placenta	Labour pains.	-	After 1.5–2 hours of bleeding the woman was transferred to the hospital by taxi.	Placenta was retained for 1.5–2 hours before transferred to the hospital,
3	Eclampsia	Generalized oedema.	Returned home and the husband made the decision to seek care again after 6 hours	Went to MCH clinic and was referred to the hospital, but went home instead and finally to the hospital.	-
4	Eclampsia and haemorrhage	Cramping. Generalized oedema.	Delivered at home by a TBA Decided to seek medical care after cramps had started.	Went to the MCH clinic, then referred to the hospital. Experienced transportation difficulties.	-
5	Haemorrhage and obstructed labour	Foetus not moving	Transport was not available at night. Not until in the morning 5 hrs. later.	Went to the local pharmacy, was referred to an ultrasound centre but this facility was closed because of mid-day break and the women returned home then went to the hospital.	Blood transfusion needed but was not readily available. Relatives and members of the public donated blood.
6	Haemorrhage	Bleeding and infection	Deliver at home by a TBA Stayed home for over two hours before deciding to seek medical care.	-	Seen at the hospital and received medicines and was then discharged and went home, then went to another health facility and received more medicines and was sent home. The women died after having returned home
7	Eclampsia	Cramping. Generalized oedema	Hospital stay was planned but escorts retuned home with the woman.	Sought care at three different facilities.	
8	Eclampsia	Cramping, generalized oedema.	Decided to seek medical care after cramps had started.	Transported from home to the MCH clinic, from where she was transferred to the hospital. Then transferred to a private hospital. These health facility had no doctors thus the woman returned home and died.	The hospital was closed and the escorts took the woman to another health facility which was also closed.
9	Haemorrhage and obstructed labour	Labour pains and bleeding.	Decided to seek medical care after labour had started and had been bleeding for more than 12 hours .	Experienced transportation difficulties in the night.	Blood transfusion was needed but was not available. Relatives and members of public had no matching blood group.
10	Eclampsia and haemorrhage	Generalized oedema.	Decided to seek medical care after she was too week to move.	Seeking care a the local pharmacy three different times.	VHW in the local pharmacy could not help.
11	Anaemia	Tired and weak	Decided to seek care when too weak to move	Seeking care from two different health centres.	No active management at the MCH clinic or at the private hospital. The woman was seen at the outpatient clinic and was given tablets and sent home.
12	Eclampsia	General oedema, blurred vision	Sought medical care after her eyesight became blurred.	-	No active management at the pharmacy and no referral to an obstetric care provider.
13	Haemorrhage, hypertension and sepsis	Bleeding.	Delivered at home by a TBA. Stayed home for 10 days before receiving medical care.	Seeking care from traditional healers. Experienced difficulties in finding money for medicines.	The health care providers; the village health worker and others did not refer the woman to a MCH clinic or to the hospital.
14	Haemorrhage, anaemia	Prolonged labour, bleeding	Sought medical care after bleeding for an hour and noticing that the baby was not positioned properly.	Sought care at the MCH clinic, then referred to the hospital but returned home and called village health worker. After deciding to seek care again transport was not available.	No active management by the VHW and no referral to an obstetric care provider.
15	Haemorrhage, anaemia	Bleeding.	Delivered at home by a TBA. Then decided to seek care because of bleeding that started during labour.	-	-
16	Haemorrhage and sepsis	Offensive vaginal discharge, fever	-	-	No active management.
17	Sepsis	Abdominal pain, fever and shivering		Experienced money and transport difficulties among the relatives who was supposed to take her to the hospital	Medicines were given at the MCH clinic, but the woman was then sent home.
18	Haemorrhage and obstructed labour	Bleeding.	Called a TBA when she had lost a large amount of blood	Experienced transport difficulties for two hours.	Was delayed at the MCH clinic for three hours.
19	Obstructed labour	Prolonged labour.	No decision was made to seek care until after10 hours in labour.	Experienced transport difficulties in the night	No active management by the TBA and no referral to obstetric care provider.
20	Haemorrhage, hypertension.	Bleeding.	Sought medical care after she turned pale.	-	Stayed 10 days in the hospital but died when she returned home.
21	Haemorrhage, obstructed labour	Prolonged labour and bleeding.	Sought medical care after many hours of bleeding		Needed blood transfusion but the escort had no money so they searched blood donors but could not find anyone with a matching blood group.
22	Eclampsia.	Generalized oedema.	-	-	Died 30 minutes after giving birth, having returning home from the hospital where she had stayed the previous 10 days.
23	Haemorrhage.	Bleeding, followed by headache and fever.		The family sought care by the community health worker.	No active management was provided by the community health worker and no referral to an obstetric care provider was made.
24	Obstructed labour, hypertension.	High blood pressure and obstructed labour	Fist delay was made by the woman’s mother in deciding to seek care only after sixteen hours of labour.	Further delay due to visits at many health providers, e.g. the Community Health Worker.	
25	Obstructed labour, haemorrhage	Prolonged labour and haemorrhage	The woman’s family decided to seek care only after thirty six hours of labour.	Long distance to health facility, took more than one hour to walk and other transportation was not available.	Ambulance was not available for the MCH
26	Anaemia, hypertension.	Hypertension anaemia, blood loss, poor appetite and weight loss.	The woman’s family decided to seek medical care only after thirty after onset of severe symptoms.		Blood transfusion needed but was not available. Relatives and members of the public donated the blood. Due to limited financial support, the women was discharged from the hospital.
27	Obstructed labour, haemorrhage	Fever, vomiting, prolonged labour and haemorrhage.	There was no first delay, the woman took a correct decision to seek care initially, but she then decided to return home after labour had decreased.		The Village Health Worker gave an injection to induce labour, which resulted in bleeding. A the decision to refer her to the hospital because of the bleeding was not made.
28	Eclampsia, obstructed labour	High blood pressure,	The woman’s oldest son and daughter refused to follow the doctor’s advice of making a caesarean section	The woman went to the MCH clinic, was then referred to the hospital, but returned home after she had refused to follow the doctor’s recommendation to make a caesarean section. The woman then called a TBA.	
29	Sepsis and haemorrhage.	Fever and bleeding.	The women waited for one week to seek medical care despite bleeding	The woman sought care at multiple health care facilities e.g. the private hospital and was then referred to the referral hospital.	The woman was admitted to the hospital, and received a blood transfusion and then she returned home due financial limitations and limited family support.
30	Sepsis.	Bleeding, developed a rash after blood transfusion	The woman decided to seek medical care only after seven days of bleeding.	The woman sought care at the hospital, then she returned home and later sought medical care again.	Woman feels sick and got a skin rash after her blood transfusion in the hospital

### Verbal autopsy and Three Delays Model

Finally, the Three Delays Phases Model was applied for the analysis of the information collected from the informants. A delayed decision to seek medical care was indicated for 25 of the cases. Twenty-two of the women had delays in reaching an appropriate obstetric care facility once the decision to seek care was made. Even when an appropriate obstetric care facility was reached, 24 out of the 30 cases had not received the care services they needed, according to the reviewers. Looking at the phase of delay, twelve of the 30 cases had all three delays; 21 cases experienced two phases of delays and two cases experienced only one type of delay. A summary of the characteristics of all of the delays experienced among the 30 cases is provided in Table 5.

#### Delay in deciding to seek health care (phase 1)

The main factor behind this type of delay, which was classified as a ‘patient factor’ identified by this study, was an underestimation of signs, symptoms, and severity of the problem. Lack of knowledge about danger signs and warnings during pregnancy and failure to seek prompt treatment from health facility were the causes of delay in deciding to seek health care. Family members such as mothers and sisters were often involved in the decision-making process. In Case 13, a sister narrated:

‘During pregnancy she complained about breathlessness at the eighth month of pregnancy, her body was swollen too. Therefore, she had to measure her blood pressure and it was found to be high. She had no medication for the diagnosed hypertension due to the difficulty in finding money for the medicines. However, we occasionally bought medicines from the local pharmacy when we could afford, and we got some medicine from MCH and also from another health centre. Eventually, she delivered a dead baby at home, and after delivery she lost a large amount of blood, I was constantly changing her bed. The bleeding subsided in the next six days. She was very weak and became pale, she had fever and headache, and her swelling increased. The family applied traditional remedies to treat her. Health providers visiting the village examined her and gave her injections and told us to take her hospital. Therefore, she told me to go to the market and sell her earrings. I sold them for $50 worth of shillings but soon after I arrived she died at home. It was on her ninth day after delivery’.

#### Delay in reaching obstetric care facility (phase 2)

Transportation difficulties and seeking care at more than two medical facilities, were contributing factors in failing to access quality emergency obstetric care. The families experienced difficulty in collecting enough money to go to another health facility to access care. One of the interviewed mothers narrated:

‘She felt that the foetus did not move for a day and she went to the local pharmacy, where she was advised to have an ultrasound. She visited the ultrasound clinic, but the clinic had closed at the time due to the daybreak at noon. She returned home and in the afternoon the labour started, The TBA was called for … . .It turned out to be a multiple pregnancy after one baby was delivered the next baby did not follow, so I walked in the heat of the sun to the MCH, but the staff told me to bring her to the clinic. … … When I came back home with a rented car she already had lost a lot of blood and died soon after’.

#### Delay in actually receiving health care after reaching the hospital (phase 3)

Inadequate health services was one of the factors in this study. This includes lack of blood as in Cases 5 and 9 and incompetence of the available staff as shown in Case 2: Placenta was retained after delivery in the health centre; however no manual removal of placenta was performed, and no timely referral was made at the health centre. The woman’s neighbour narrated:

‘After she experienced labour pain she decided to go to the health centre. Her daughter and I accompanied her to the centre two hours later when her contraction became more intense. A friend with a car helped us to take her there and her aunt was left home to babysit. After the nurse had examined her she told us that her cervix was dilated 4 cm. So she stayed to deliver there. Finally, she delivered a baby girl but the placenta was not coming out. Immediately she told the nurse that in her previous pregnancy the placenta was also retained first and then manually removed. However, this was not attempted. Consequently, she was bleeding heavily. The midwife gave her an injection to stop the bleeding. After two hours when the dawn broke she was transferred to the hospital. After 20 minutes in the hospital the doctors told us that she was dead’.

Other inadequate health services factors included missed diagnoses such as anaemia (Case 11), tumour (Case 16), and multiple pregnancies, which were not identified during their ANC visits. Another factor was mismanagement of intra and postpartum haemorrhage by a TBA delaying timely referral (Cases 18 and 19). Case 10 who made no antenatal care visits but instead visited the pharmacy after complications, where medicine was given but no referral was made. Case 11 made five ante-natal care visits but had to queue for seven consecutive days at the health centre because of a delay in receiving a registration for vouchers. Delay in accessing health services may have been experienced as a result of perceived poor quality of care by the families: some families testified about health staff’s mistreatment towards escorts in addition to limited privacy and lack of personal care at the delivery facility.

Some patient factors were also identified. For instance, refusal to accept care (Case 3, 14 and 20) because of fear of caesarean section. Furthermore, patient factors often contributed to discharge from a health facility without complete treatment. One woman (Case 20) went home from the hospital after 10 days because of financial constraints and another woman (Case 7) made the same decision, because of childcare responsibilities. However, in some cases, the health staff made the decision to discharge the woman without complete treatment (Case 6, 11 and 17) giving the women medicine to take home.

## Discussion

In Somalia, the lifetime risk for a woman to die because of pregnancy is estimated to a staggering 5%, which makes maternal mortality a major contributor to the Burden of Disease among adult women in this country. Moreover, maternal deaths often have detrimental effects on family health and wellbeing, in a low-income country context where child-rearing responsibilities rest heavily on the woman in the household. This detailed VA-based study of 30 cases of maternal deaths in Bosaso District, which exhibited the typical direct and indirect obstetric causes of death, has broadened understanding of the socio-cultural and health systems-related factors behind maternal mortality.

The majority of the deaths were direct obstetric deaths due to haemorrhage, followed by eclampsia, obstructed labour and sepsis. This is consistent with findings of other studies including a WHO global estimate [1, 6, 14–16]. One study from Mozambique and some other African countries identified sepsis as the second leading direct cause of death and HIV/AIDS was the most common indirect cause of death [17].

The main finding in this study was that all three phases of delay were frequent, often in parallel.

### Delay in decision to seek health care (first delay)

A combination of factors contributed to the first delay, which lead the pregnant women not to seek prompt treatment from a health facility with capacity to manage pregnancy and delivery-related complications. Some mothers did nothing in this situation, while others sought treatment from TBAs (as the first place to seek care which is in accordance with a study from Gambia [18]) or Village Health Workers (VHWs), while other purchased medicine from the pharmacy or sought treatment by spiritual healers.

Obstetric emergency among the studied cases, occurred mostly during the intrapartum or immediately postpartum. In this study 19 of the women delivered at home increasing the risk of maternal mortality from delays.

Traditional birth attendants played a major role in this study as health service providers. Delay in referral from a TBA to a health facility with emergency obstetric care and skilled birth attendants can result in a high risk of maternal death [19]. For example, the six cases in this study where sepsis was either direct or underlining cause of death, were all delivered at home assisted by a TBA. It has been shown in other studies that sepsis usually follows haemorrhage in maternal deaths [12] and previous studies have shown that facility-based delivery can reduce the risk of maternal death [15]. It has also been shown in a study of maternal near miss cases in Brazil, that delay in accessing emergency obstetric care increases the severity of the complications [6].

### Delay in reaching health care once decided (second delay)

Contributing factors for not reaching an appropriate health facility were often transport issues or financial constraints. Other factors were lack of doctors on duty, causing the women to move from one facility to the next in desperation for getting adequate service. Further delay in reaching care may have been caused by the perceived poor quality of care by the families. Some families in this study reported experiences of health personnel’s mistreatment towards escorts. Moreover, they mentioned limited privacy and lack of personal care at the delivery facility.

### Delay in receiving prompt and adequate care once in health facility (third delay)

Once the woman had reached the hospital, in some cases she did not receive prompt and adequate treatment due to both patient and health worker factors. A previous systematic review of third delay studies [20] has shown that women are at risk of dying even if they arrive in the health facility without any complications [20–22]. Other studies reviewing mortality audits also found health service factors to be common contributing factors [20–22] Our finding of inadequate health service factor is consistent with their results. A recent study performed in Bosaso health facilities assessing the essential newborn care supports the finding of this study of poor quality of obstetric care [23].

In the cultural setting represented by Somalia, women are expected to take full responsibility for childcare duties and all of the domestic chores, regardless of other circumstances. In addition, many of them often have the obligation to help supporting the family through poorly paid jobs or by selling items in the market, even during pregnancy. Almost half of the women in the study worked for income. Moreover, almost all of their jobs were in the informal/labour sector. Hard work and responsibilities coupled with financial constraints during pregnancy might have been a reason for women overlooking their health status and not utilizing facility based obstetric services such as ultrasound.

### Methodological considerations

A strength of this study was that the VAQ was piloted to identify issues that could improve validity. A further strength was that agreement regarding cause of death between the three reviewers was 100%. However, a limitation was the absence of information regarding women who survived their delivery experience, which might have given more clarity to the actual prevalence of risk factors of maternal emergencies. Another limitation was the small sample size because of resource constraints, meaning that the results cannot be generalized. The study should, therefore, be regarded as mainly exploratory.

We observed that family members or people in the community felt more comfortable about talking if the interviewer was accompanied by someone they knew. For that reason, the investigator was accompanied by a health staff person (nurse or midwife) from the nearby MCH, or by a TBA or a VHW. However following introductions the accompanying person left so that the interviewer could proceed with the interview. One of the challenges was that the tracing and retrieval of health facility patient records was at times difficult. Some records were missing, incomplete and lacked clarity regarding key information.

## Conclusion

Haemorrhage, eclampsia, sepsis and obstructed labour were important causes of direct obstetric deaths. Anaemia was a risk factor behind the indirect obstetric deaths. Patient factors such as delaying the decision to seek care when obstetric complications arise and health service factors were identified as common contributors to maternal death.

There should be national policies or protocols put in place to track and review all maternal deaths. Furthermore, Health Demographic Surveillance Sites utilising VA techniques could be initiated to monitor and audit cases of maternal mortality. Institutional delivery is the key to reducing high maternal mortality and pregnant women need to access adequate delivery facilities as soon as labour starts.

## Paper context

There is a lack of systematic studies exploring the ‘road to death’ of maternal deaths in Somalia, a country without vital registration. This study analysed the background factors and determinants of maternal mortality using an adapted VA approach based on the ‘Three Delay Model’. Delays were often identified in the maternal deaths resulting from sociocultural, economical and health service factors obstructing access to adequate and prompt health care. This study adds important new knowledge about contextual factors behind maternal mortality in Somalia.
